# A perspective on the use of the cervical flexion rotation test in the physical therapy management of cervicogenic headaches

**DOI:** 10.1186/s40945-022-00153-2

**Published:** 2022-12-08

**Authors:** Jean-Philippe Paquin, Jean-Pierre Dumas, Thomas Gérard, Yannick Tousignant-Laflamme

**Affiliations:** 1 Laboratoire de Recherche Biomécanique et Neurophysiologique en Réadaptation Neuro- musculosquelettique(BioNR), Université du Québec à Chicoutimi (UQAC), G7H 2B1 Saguenay, Qc Canada; 2 Department of health sciences, Université du Québec à Chicoutimi (UQAC), G7H 2B1 Saguenay, Qc Canada; 3 School of Rehabilitation, Faculty of Medicine and Health Sciences, Université de Sherbrooke, 3001 12e Avenue Nord, J1H 5N4 Sherbrooke, QC Canada; 4 Research Center of the CHUS (CRCHUS), Centre hospitalier universitaire de Sherbrooke (CHUS), Sherbrooke, Qc Canada; 5 Cabinet de kinésithérapie, 7 rue Antigna, 45000 Orléans, Loiret, France

**Keywords:** Cervicogenic headache, Physiotherapy, Cervical flexion-rotation test, Diagnostic, Validity

## Abstract

**Background:**

The Cervical Flexion-Rotation Test (CFRT) is widely used in the assessment of upper cervical spine mobility impairments and in the diagnosis of cervicogenic headache (CGH) by physiotherapist. Many studies investigated its different properties, and the results show that the CFRT has good construct validity in the measurement of C1-C2 rotation as well as good to excellent reliability.

**Purpose:**

In this theoretical paper, we explore the value and point out two methodological issues associated to the CFRT, one related to the procedures and another related to its diagnostic accuracy.

**Results:**

Our analysis indicate that there are many confounding factors that could affect the CFRT cut-off’s accuracy, which are likely to overestimate the diagnosis properties of CFRT. Potential solutions are discussed. Moreover, the gold standard (manual examination) used to examine the validity of the CFRT for the diagnosis of CGH appears to be far from perfect - we could argue that the diagnostic properties of the CFRT for CGH might be biased and the likelihood ratios are likely to be overestimated. However, it could be relevant to explore if results of the CFRT could be considered as a treatment-effect modifier. Maybe the CFRT could be more valuable as a prognostic factor?

**Conclusion:**

The quality of evidence supporting the validity of the CFRT is most likely biased. In the absence of a better gold standard, maybe the CFRT could be a more valuable test to establish the patient’s prognosis and help the clinician to choose the most appropriate treatment options.

## Introduction

With prevalence estimates ranging from 0.17 to 4.1% in the general population [1], *cervicogenic headache* (CGH) is defined as a secondary headache (HA) resulting from a disorder involving any bony of soft-tissue structure of the neck [2] and represents between 15 and 20% of all chronic HAs [2]. Although the exact underlying mechanisms are not fully understood, evidence shows that CGH could arise from the C1-C2 zygapophyseal joints [3]. As a result, one of the main clinical features of CGH is the loss of mobility during active cervical range of motion (ROM) and hypomobility of the upper cervical spine [2]. This clinical feature is used as a diagnostic criterion by the Cervicogenic Headache International Study Group (CHISG) [2].

Movement deficits specific to the upper cervical spine can be assessed using the cervical flexion-rotation test (CFRT) [4], a test that can isolate the rotation movement of the upper cervical spine. Magnetic resonance imaging made it possible to document that a 45^o^ rotation on both side is considered as “normal” range of motion during the CFRT [4].

As the CFRT demonstrated good validity in the assessment of upper cervical spine mobility deficits [4], it can be used to support the diagnosis of C1-C2 related CGH. However, as the CFRT informs about movement impairment (versus pain provocation), could it be useful for other purposes, such as treatment guidance, particularly for physical rehabilitation approaches? Yet, before answering this question, we would like to point out two issues associated to the CFRT, one related to the procedures (cut-off) and the second one related to its diagnostic accuracy. Some potential solutions are also proposed and discussed.

### Issue n°1: the precision of the cut-off for positive response

The CFRT test is considered positive only when movement restrictions are present. However, there are inconsistencies in the cut-off (ROM) to rule on this movement restriction. Yet, significant confounding factors may increase the risk of bias or misinterpretation of CFRT.

1) Influence of pain: performing the CFRT during a painful HA episode might alter the movement response, as pain can clearly inhibit or limit ROM.

2) Age: it was established that CFRT range of motion decreases with age, as 27.9% of the variance of the CFRT could be explained by age alone [5].

3) Assessment tool: Although eyeball estimation of cervical rotation movement makes the CFRT very “clinician-friendly”, it has measurement errors that might greatly affect the interpretation of the test [6].

4) Misdiagnosis: Positive CFRT could be found in migraine population. Moreover, chronicization of symptoms increases the rate of positive CFRT in this population. This can lead to misdiagnosis [3].

Markedly, these confounding factors could affect the cut-off’s accuracy and is likely to overestimate the diagnosis properties of CFRT.

Potential solutions to reduce the influence of these confounding factors include:

#### 1) Promote the use of appropriate measurement devices to enhance measurement precision

We believe that using a valid instrument to measure neck rotation during the CFRT may (i) increase the inherent validity and reliability of the test, (ii) help clinicians to detect more subtle loss of movement and (iii) bring clinically relevant information, to assist the diagnosis and to monitor significant improvements in range of motion following an intervention. Different devices measuring neck range of motion have been validated in previous studies and could be used to measure cervical range of motion while performing the CFRT. These instruments include the CROM device, smartphones applications and, more recently, the *EasyAngle* (Meloq AB, Stockholm, Sweden) digital goniometer, which have been used to measure ROM during the CFRT [7]. Clearly, the use of a validated tool is essential to minimize measurement error.

#### 2) Consider integrating the symptomatic response to the decision process

As pain is the main symptom of CGH, where the HA can be caused by an impairment of C1/C2 zygapophysial joints, it would be reasonable to assume that the CFRT should reproduce the patient’s typical symptoms (headache). Yet, we found that the reproduction of the patient’s typical pain was used in only one study [8]. The addition of this criteria to the decision process may increase the accuracy of CFRT.

## Diagnostic properties of the CFRT

The diagnostic properties of the CFRT have been studied and evidence show that the CFRT can be used to support the diagnosis of C1-C2 related CGH [5]. Moreover, the CFRT can be used to differentiate the diagnosis of CGH from migraine [3].

The studies that investigated the diagnostic properties of the CFRT used manual examination as the gold standard. A systematic review found that the CFRT is the most reliable and accurate for CGH [9].

Although these findings are most likely reassuring about CFRT’s clinimetric proprieties, only one concern remains: the gold standard used in these validation studies.

### Issue n°2: the CFRT’s gold-standard

Most studies pertaining to the diagnostic properties of CFRT used expert-derived consensus criteria that considered manual examination, which incorporates passive physiological and accessory intervertebral movement tests by an experienced clinician (manual therapy skills). Hypomobility and/or pain reproduction observed during manual examination serves as the “gold standard” to diagnose C1-C2 related CGH.

Yet, data from studies who investigated reliability of manual examination shows notable variability (κ = -0,05 to 0,86) and poor reliability (κ = 0,28) [10]. Furthermore, studies reporting variability and reliability had high risk of bias according to the authors of the systematic review, with a score of 6/11 on the quality appraisal of reliability studies (QAREL) checklist, mainly for blinding reasons [8]. As described by Castien et al. in 2015 [11], the inconsistencies in the reliability of manual examination of C1-C2 and the absence of details regarding the specific gold standard as the reference test used in other CFRT validity studies, we could argue that the diagnostic properties of the CFRT for CGH might be biased - the likelihood ratios currently reported are likely to be overestimated.

On the other hand, what else could be used as gold standard to study the CFRT’s diagnostic properties? Evidence shows that the best available gold standard to confirm C1-C2 related CGH are zygapophyseal joints nerve blocks [2]. However, this technique is quite invasive, rarely accessible and has inherent risks [2]. This lack of proper gold-standard leads to an unsolvable problem to validate the CFRT, whose true results are only approximations.

Potential solution: *Consider the CFRT as a prognostic factor versus a diagnostic test?*

As the gold-standard used to establish the CFRT’s diagnostic accuracy poses many challenges, it could be relevant to explore if a positive response of the CFRT (reduced upper cervical range of motion + reproduction of symptoms) could be considered as a treatment-effect modifier. Treatment-effect modifiers are characteristics of a person that predict a response to a treatment. They influence the relationship between a specific intervention and an outcome and have the potential to guide clinical decision-making for disease management.

Prognosis is a promising approach that can be used to better tailor the treatment according to the patient’s profile. The magnitude of range of motion loss in the upper cervical spine might also help in determining prognosis. There is an association between upper cervical flexion and extension and neck-related disability, HA frequency and intensity in neck pain patients [12]. It is fair to assume that “normalizing” upper cervical range of motion (thus, bring back CFRT range of motion to normal values) could help reduce HA severity.

For example, as the first line of treatment for CGH includes manual therapy techniques and exercises, such as SNAGs (Sustained Natural Apophyseal Glides) and self-SNAGs on C1-C2 level [2]. Thus, a positive CFRT could be a prognostic factor, which could support the indication for the application of these techniques to reduce pain or disability associated to CGH. Hence, we argue that positive CFRT, defined by the reproduction of well-known pain and/or a decrease in ROM during the test could be seen as a treatment-effect modifier rather than a diagnostic tool for C1-C2 dysfunction. Accordingly, the CFRT would be a marker that could define the usefulness of a given rehabilitation technique.

Nevertheless, the validation of a prognostic factor or model is a complex process. It requires the use of cohorts to develop and test the internal/external validity of the model. These validation steps, unlike the validity of a diagnostic tool, do not require a gold standard. Future well-conducted studies are therefore relevant to define whether the results of the CFRT could be used as a prognostic factor or integrated into a prognostic model.

## Conclusion

The CFRT is widely used by rehabilitation clinicians in the assessment of patients suffering of HA to help with the diagnosis, but also to detect upper cervical spine mobility impairments in relation to the symptoms. Considering the growing evidence of upper cervical spine mobility impairment in different pathologies such as cervicogenic dizziness, post-concussion syndrome and temporomandibular disorders, and evidence of benefits of addressing cervical spine impairments in patients with such pathologies, the CFRT might be an interesting test to assess C1-C2 mobility in these populations.

However, we have to keep in mind that the gold standard used to examine the validity of the CFRT for the diagnosis of CGH is not perfect - most studies used manual examination procedures, which have moderate reliability at best. Furthermore, the fact that different cut-off values are found in the literature can be confusing – considering many cut-off values are reported, which one should clinicians used to determine a positive CFRT? How painful symptoms or aging might affect the cut-off? It may be relevant (and comforting) to consider that the CFRT could be a more valuable test to establish the patient’s prognosis and help the clinician to choose the most appropriate treatment options.

## Data Availability

Not applicable.

## List of abbreviations

CFRT: cervical flexion rotation test
CGH: cervicogenic headache
HA: headache
ROM: range of motion

## References

[1] Knackstedt H, Bansevicius D, Aaseth K, Grande RB, Lundqvist C, Russell MB (2010). Cervicogenic headache in the general population: the Akershus study of chronic headache. Cephalalgia.

[2] Verma S, Tripathi M, Chandra PS (2021). Cervicogenic Headache: Current Perspectives. Neurol India.

[3] Oliveira-Souza AIS, Florencio LL, Carvalho GF, Fernández-De-Las-Peñas C, Dach F, Bevilaqua-Grossi D (2019). Reduced flexion rotation test in women with chronic and episodic migraine. Braz J Phys Ther.

[4] Takasaki H, Hall T, Oshiro S, Kaneko S, Ikemoto Y, Jull G (2011). Normal kinematics of the upper cervical spine during the Flexion–Rotation Test – In vivo measurements using magnetic resonance imaging. Man Therap.

[5] Schäfer AGM, Schöttker-Königer T, Hall TM, Mavroidis I, Roeben C, Schneider M, et al. Upper cervical range of rotation during the flexion-rotation test is age dependent: an observational study. Therapeutic Advances in Musculoskeletal Disease. 2020 Jan 31;12:1759720X2096413. 10.1177/1759720X20964139 PMC7607754 33193833

[6] Schäfer A, Lüdtke K, Breuel F, Gerloff N, Knust M, Kollitsch C, et al. Validity of eyeball estimation for range of motion during the cervical flexion rotation test compared to an ultrasound-based movement analysis system. Physiotherapy Theory and Practice. 2018 Aug 3;34(8):622–8. 10.1080/09593985.2017.1423523 29308957

[7] Luedtke K, Schoettker-Königer T, Hall T, Reimer C, Grassold M, Hasselhoff-Styhler P, et al. Concurrent validity and reliability of measuring range of motion during the cervical flexion rotation test with a novel digital goniometer. BMC Musculoskeletal Disorders. 2020 Dec 11;21(1):535. 10.1186/s12891-020-03525-6 PMC7422569 32781990

[8] Hall T, Robinson K (2004). The flexion–rotation test and active cervical mobility—A comparative measurement study in cervicogenic headache. Man Therap.

[9] Rubio-Ochoa J, Benítez-Martínez J, Lluch E, Santacruz-Zaragozá S, Gómez-Contreras P, Cook CE (2016). Physical examination tests for screening and diagnosis of cervicogenic headache: A systematic review. Man Ther.

[10] Jonsson A, Rasmussen-Barr E (2018). Intra- and inter-rater reliability of movement and palpation tests in patients with neck pain: A systematic review. Physiother Theory Pract.

[11] Castien RF, De Hertogh W, Scholten-Peeters GGM. Letter to the Editor: Physical examination tests for screening and diagnosis of cervicogenic headache: A systematic review by Rubio-Ochoa et al. (2015). Man Ther. 2016 Jun;23:e7-8. 10.1016/j.math.2016.01.006 26934859

[12] Ernst MJ, Crawford RJ, Schelldorfer S, Rausch-Osthoff AK, Barbero M, Kool J (2015). Extension and flexion in the upper cervical spine in neck pain patients. Man Therap.

